# Towards better reliability in fetal heart rate variability using time domain and spectral domain analyses. A new method for assessing fetal neurological state?

**DOI:** 10.1371/journal.pone.0263272

**Published:** 2022-03-01

**Authors:** Anne Rahbek Zizzo, Ida Kirkegaard, Niels Uldbjerg, John Hansen, Henning Mølgaard

**Affiliations:** 1 Department of Obstetrics and Gynecology, Aarhus University Hospital, Aarhus N, Denmark; 2 Department of Health Science and Technology, Aalborg University, Aalborg, Denmark; 3 Department of Cardiology, Aarhus University Hospital, Aarhus N, Denmark; University of Washington, UNITED STATES

## Abstract

**Objectives:**

Fetal heart rate variability (FHRV) has shown potential in fetal surveillance. Therefore, we aimed to evaluate the reliability of time domain and spectral domain parameters based on non-invasive fetal electrocardiography (NI-FECG).

**Method:**

NI-FECG, with a sampling frequency of 1 kHz, was obtained in 75 healthy, singleton pregnant women between gestational age (GA) 20^+0^ to 41^+0^. The recording was divided into a) heart rate pattern (HRP) and b) periods fulfilling certain criteria of stationarity of RR-intervals, termed stationary heart rate pattern (SHRP). Within each recording, the first and the last time series from each HRP with less than 5% artifact correction were analyzed and compared. Standard deviation of normal-to-normal RR-intervals (SDNN), root mean square of successive differences (RMSSD), high frequency power (HF-power), low frequency power (LF-power), and LF-power/HF-power were performed. A multivariate mixed model was used and acceptable reliability was defined as intraclass correlation coefficient (ICC) ≥ 0.80 and a coefficient of variation (CV) ≤ 15%. Based on these results, the CV and ICC were computed if the average of two to six time series was used.

**Results:**

For GA 28^+0^ to 34^+6^, SDNN and RMSSD exhibited acceptable reliability (CV < 15%; ICC > 90%), whereas GA 35^+0^ to 41^+0^and 20^+0^ to 27^+6^ showed higher CVs. Spectral domain parameters also showed high CVs However, by using the mean value of two to six time series, acceptable reliability in SDNN, RMSSD and HF-power from GA 28^+0^ was achieved. Stationarity of RR-intervals showed high influence on reliability and SHRP was superior to HRP, whereas the length of the time series showed minor influence.

**Conclusion:**

Acceptable reliability seems achievable in SDNN, RMSSD and HF-power from gestational week 28. However, stationarity of RR-intervals should be considered when selecting time series for analyses.

## Introduction

Heart rate variability (HRV), in the form of time domain and spectral domain analyses, reflects the autonomic nervous system in adults [1], neonates [2, 3] and fetuses [4, 5]. Clinically this feature of fetal heart rate variability (FHRV) may be valuable regarding fetal surveillance. Thus, impaired fetal heart rate variability (FHRV) has been associated with both fetal and neonatal conditions, such as fetal growth restriction (FGR), fetal acidosis during labor, neonatal sepsis, and hypoxic ischemic encephalopathy (HIE) [6–9]. The existing fetal surveillance in high-risk pregnancies is primarily based on fetal Doppler ultrasound and reflects the cardiovascular capacity of the fetus but to a lesser extent fetal neurological function. FHRV may fill this gap in the existing surveillance. Information from both modalities could potentially provide more differentiated insight into fetal wellbeing.

Time domain and spectral domain analyses, both measurements of beat-to-beat heart rate variability, require an accurate detection of every single normal heartbeat, which conventional cardiotocography (CTG) cannot provide. Therefore, recent developments in non-invasive fetal electrocardiography (NI-FECG), where fetal ECG is obtained by electrodes placed on the maternal abdomen, has led to optimism about a clinically feasible method to monitor fetal FHRV.

Unfortunately, previous work has failed to address the reliability of FHRV. Most likely this reliability is affected by several factors known to affect HRV. In adults, these factors include length of analyzed time series and correction of artifacts of inter-beat intervals (RR-intervals), caused by noise, ectopic and missing beats, non-normal beats (ectopic beats) etc. Other important factors are the stationarity of the mean RR-interval and the autonomic nervous system (ANS), which in adults are sought to be controlled by a standardized setup including a resting position and controlled breathing during ECG acquisition. In fetuses these factors are difficult to control. However, the fetal heart rate pattern (HRP) [10], which reveals the fetal behavioral state at least when applied on fetuses after GA 35 weeks [11], might be useful in regard to ensuring the stationary activity of ANS. Many studies restrict FHRV analyses to certain HRP, but whether the stationarity of mean-RR is also ensured is often not stated [10, 12–14]. Gestational age (GA) and likely maternal smoking as well are also important factor affecting FHRV [15, 16], while fetal gender [17, 18] and ethnicity [19] are likely to have a minor influence on FHRV.

The aim of this study is to assess the reliability of time domain and spectral domain FHRV parameters in healthy fetuses based on NI-FECG. The potential impact of gestational age, length of time series and method used for selection of time series on reliability is also evaluated.

## Methods

### Population

At the regional hospital in Horsens, Denmark, we included healthy singleton pregnant women who provided their informed and written consent. All women were included at one of the routine and scheduled midwife consultations. The included women attended the national, prenatal screening program. The gestational age was determined by the means of crown rump length (CRL) using the formula of Robinson et al [20] between GA 8^+0^–13^+6^. Exclusion criteria were fetal malformations, multiple pregnancy, obstetric complications, maternal chronic disease, and signs of labor including regular contractions. The study was approved by the Danish Data Protection Agency [1–16–2–440–15] and the Danish National Committee on Health Research Ethics [1–10–72–227–15].

The participants were divided into three groups in accordance to their gestational age (GA): group A) 20^+0^–27^+6^; group B) 28^+0^–34^+6^; group C) 35^+0^–41^+0^. This classification was based on 1) the isolating effect of vernix caseosa, which is particularly challenging in gestational weeks 28–34, so we wanted to assess the performance of NI-FECG in that specific period; 2) fetal behavioral states (FBS) [21], which are closely related to movements from gestational week 35 onwards [11], so heart rate pattern (HRP) [10] might perform better in these weeks compared to the former weeks (see section “Selection of time series”); and 3) the cut-offs, which seem reasonable when considering treatment and intervention in complicated pregnancies, as gestational weeks 28 and 34 are important milestones in the perspective of neonatal outcome.

### Acquisition of NI-FECG

**Fig 1:**
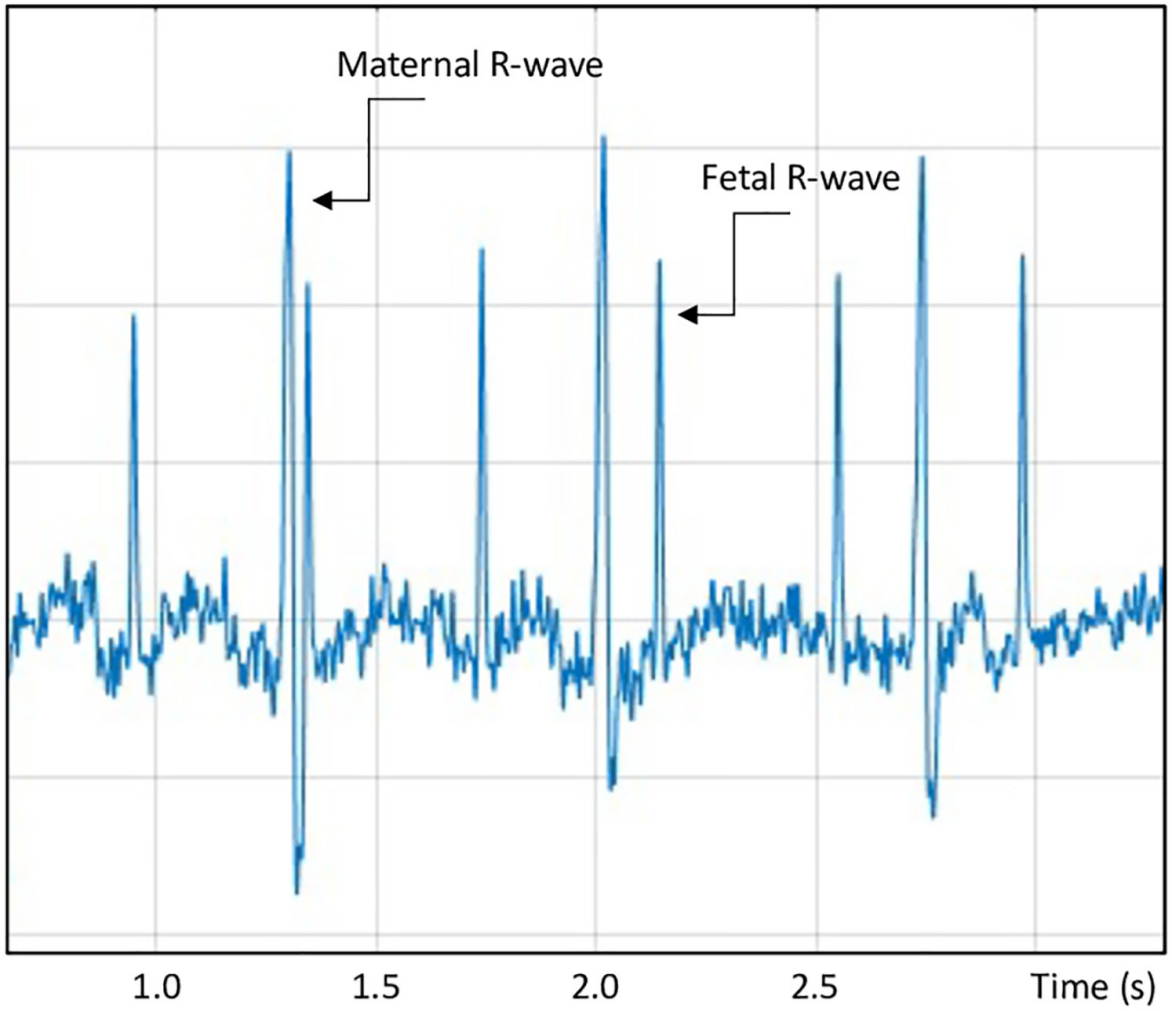
Non-invasive fetal electrocardiogram (NI-FECG) showing both maternal and fetal R-waves.

NI-FECG was obtained by four electrodes (Ag/AgCl) and one ground electrode placed on the maternal abdomen and connected to a fetal ECG-recorder (Viewcare A/S, Søborg, Denmark). The pregnant woman was placed in a supine or lateral resting position in a quiet room. At least 20 minutes of FECG was obtained. Each acquisition was performed between 8AM and 4PM with a 24-bit resolution and a sampling frequency of 1 kHz. High and low pass filters were applied at 2 Hz and 150 Hz to remove DC offsets and noise. The cut-off frequencies were chosen in order to minimize motion artifacts, reduce noise and keep the information related to beat detection. A notch filter at 50 Hz was applied to remove any possible power line contamination. The algorithm for automatic fetal R-wave detection was based on templates of fetal and maternal complexes and was developed by Viewcare A/S (Fig 1) [22].

### Processing

The software package used to perform the FHRV analyses was Kubios Premium (Kubios heart rate variability software version 3.3; Biosignal Analysis and Medical Imaging Group, Department of Physics, University of Kuopio, Kuopio, Finland). By using Kubios, detrending was performed based on Smoothen Priors Regulations [23]. The method works like a time-varying high pass filter, smoothing the data according to the value of the smoothing parameter, which was set to 500 corresponding to a cut-off frequency of 0.035 Hz [24]. All of the time series were systematically and manually checked for missing beats, ectopic beats, and errors in the automatic R-wave detection. Moreopver, in Kubios, artifacts were removed and replaced by Cubic Spline interpolation [24–26]. In most recordings the threshold for artifact correction was set to 40 milliseconds, meaning that RR-intervals deviating more than 40 milliseconds from the preceding RR-interval were removed and interpolated by Cubic Spline. All included segments were checked at the EKG level to ensure that only artifacts were corrected and replaced. In a few recordings a threshold of 100 milliseconds was needed to ensure that only artifacts and not true RR-intervals were corrected. To ensure evenly sampled time series, RR-intervals were re-sampled at 4Hz. This is an appropriate sampling rate for the study of autonomic regulation since it enables us to compute reliable spectral estimates between DC and 1 Hz [27]. Resampling was only applied for spectral analysis.

Recordings containing at least two three-minute epochs of good quality FECG, defined by less than 5% corrected RR-intervals and less than five successive corrected RR-intervals, were included in the analyses.

### Length of time series

Within each recording of approximately 20 minutes duration, we selected two time series (short time segments used in the FHRV analyses) of 64 seconds and 120 seconds, each of which fulfilled the classification criteria given below. These lengths were chosen based on the literature [28–30] and several factors:

The Task Force recommendations from 1996 stated that analyzed time series should be at least 10 times the wavelength of the frequency band of interest [1]. As the HF frequency-band in fetuses is higher than in adults, due to the higher frequency of respiratory movements, we assumed that short time series were appropriate. In addition, the fetal heart rate is at least twice that seen in healthy adults. This leads to extra RR-intervals per time series and thereby an increased chance of identifying “the melodies” in the continues change of RR-intervals in short time series. Nevertheless, when using 64-second time series some of the lower frequencies of the LF band might be underestimated.

Nevertheless, physiologically stable conditions are quite difficult when it comes to fetuses especially before gestational week 26, where quiet periods without movement are almost absent [11]. In later gestational weeks, longer periods of quiescence are seen, but still intermittent breathing movements remain unpredictable and might influence FHRV. By using these short time series, the chance of a “physiologically stable condition” during the analyses is enhanced. Finally, short time series permit strict criteria in terms of artifact correction and stationarity in mean RR, which is also very important in terms of achieving “controlled laboratory conditions”.

### Classification of time series

RR-intervals were converted to beats per minute (bpm) and based on a CTG-like fashion; the recording was classified both into 1) one of the three heart rate pattern (HRP), as defined by Schneider et al [10] and 2) a fourth heart rate pattern termed stationary heart rate pattern (SHRP) (Table 1). The criteria for HRP depends on GA and HRP I is classified only for GA 24^+1^ to 32^+0^ and HRP III for GA 32^+1^ to 41^+6^; therefore, these HRP were only used in these specific weeks. The criteria of SHRP were developed from a signal theoretical perspective, based on the assumption that stationarity in the mean RR is essential in the aspect of reliability of FHRV analyses on a short time segment (Table 1). SHRP was categorized by no accelerations or decelerations (± 15 bpm in 15 seconds) and floating of baseline less than 10 bpm per 2 minutes.

**Table 1:** Classification of heart rate pattern used in the selection of time series.

	SHRP^a^	HRP^b^		
		HRP I (GA 24^+1^–32^+0^)	HRPII (GA 24^+1^–41^+6^)	HRPIII (GA 32^+0^–41^+6^)
Fetal heart rate	No restriction	Fetal heart rate < 160 bpm	Fetal heart rate <160 bpm. However, fetal heart rate may exceed 160 bpm during acceleration	Fetal heart rate may exceed 160 bpm.
Floating of baseline	<10 bpm/2min	< 10 bpm/ 3 min	Unstable fetal heart rate with variant floating baseline.	Unstable floating baseline
Baseline bandwidth	No restriction	Oscillations < ± 5 bpm	Oscillations > ± 5 bpm	Oscillations > ± 10bpm
Accelerations/decelerations	Not allowed	Isolated accelerations	Frequent accelerations ***especially after GA 32***^***+0***^	Frequent, long-lasting accelerations

^a^ Stationary Heart Rate Pattern (SHRP)

^b^ Heart Rate Pattern by Schneider et al (HRP) [10]

^c^ Accelerations and decelerations defined as ±15 bpm from baseline for at least 15 seconds

#### Selection of time series

**Fig 2:**
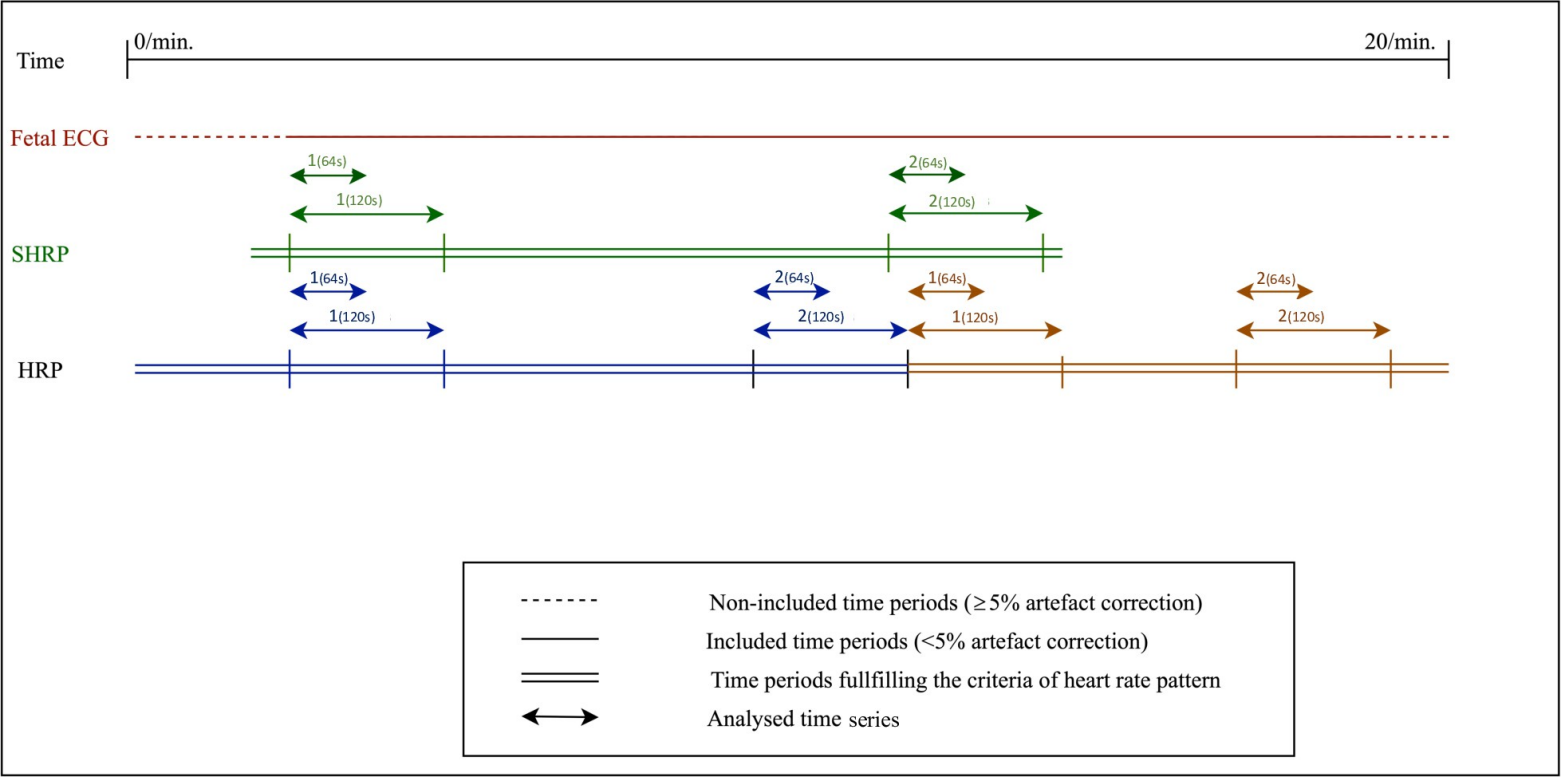
An example of the selection of time series, where time series 1 and 2 within each heart rate pattern were compared according to reliability. Fetal ECG: Non-invasive fetal electrocardiography, SHRP: Stationary heart rate pattern, HRP: Heart rate pattern [10].

Two time series from the same classification were selected in each recording and pairwise compared. To reduce the risk of information bias, the first and last time series, meeting both the criteria of signal quality and one of the tree classifications of HRP, were compared (Fig 2). However, in addition to these criteria, the time series containing the highest stationarity were compared in SHRP analyses. Some recordings contained usable time series from more than one classification of heart rate pattern. In those, time series from each classification were included in the analyses of reliability. The process of selecting the time series was blinded to the results of FHRV.

Throughout this paper, we use the terms heart rate pattern (no abbreviation) when mentioning the heart rate pattern in general, HRP when mentioning the heart rate pattern defined by Schneider et al., and SHRP when mentioning the stationary heart rate pattern, all assessed by CTG or a CTG-like fashion.

### Analyses of fetal heart rate variability

Time domain analyses consisted of the standard deviation of normal to normal RR-intervals (SDNN (ms)), and the root mean square of successive RR-interval differences (RMSSD (ms)) [24]. Spectral analyses consisted of high frequency power (HF-power (ms^2^)) and low frequency power (LF-power (ms^2^)) and LF-power/HF-power [24]. Both Fast Fourier Transformation (FFT) (without window overlap) and the autoregressive (AR) model (with an AR model order set to 24) were performed [24]. The frequency-bands were set in accordance to previous reported intervals [28, 29, 31, 32], based on the fetal respiratory rate [33]: LF-power (0.04–0.4 Hz), HF-power (0.4–1.5 Hz).

### Statistics

All parameters showed non-normality, and the variance seemed to increase with the mean (heteroscedasticity) [34]; both tendencies were most evident in the spectral analyses. After log-transformation using the natural logarithm, the data were normally distributed and homoscedastic.

Using a multivariate mixed model, assuming the same standard deviation (SD, σ) in each group of selected time series (Group 1 and 2, Fig 2), we estimated the coefficient of variation (CV) and intraclass correlation coefficient (ICC) on the log scale ((σ_B_^2^/(σ_B_^2^+σ_E_^2^)) where σ_B_^2^ denotes the between subject variance and σ_E_^2^ the within subject random error). Bland-Altman plots were carried out to identify systematic bias of the within variance (random error (E)) by estimating the mean differences between A and B and the 95% limits of agreement (LoA) on the log-transformed data. After back-transforming the data. estimates of the median ratio and 95% LoA of ratios were obtained.

Random error of the within fetus variance was evaluated by both relative reliability (ICC) and absolute reliability (CV and LoA) [34]. ICC was defined as poor (ICC < 0.4), moderate (0.4 ≤ ICC < 0.6), good (0.6 ≤ ICC < 0.8), and excellent (ICC ≥ 0.8) Based on former studies, acceptable reliability was interpreted as a CV ≤ 15% and an ICC ≥ 0.8 [34–38]. LoA complemented the interpretation by revealing the range within which 95% of the ratio (A/B) were expected to lie due to pure random variation.

The same analyses were also performed for the three gestational age groups (A, B and C).

For each gestational age and under the assumptions in the mixed model, we computed the PI, CV and ICC within each fetus if the average of two to six time series are used as measurement. These statistics only rely on the results from the two selected time series, thereby assuming the same variance also in the additional time series.

## Results

**Fig 3:**
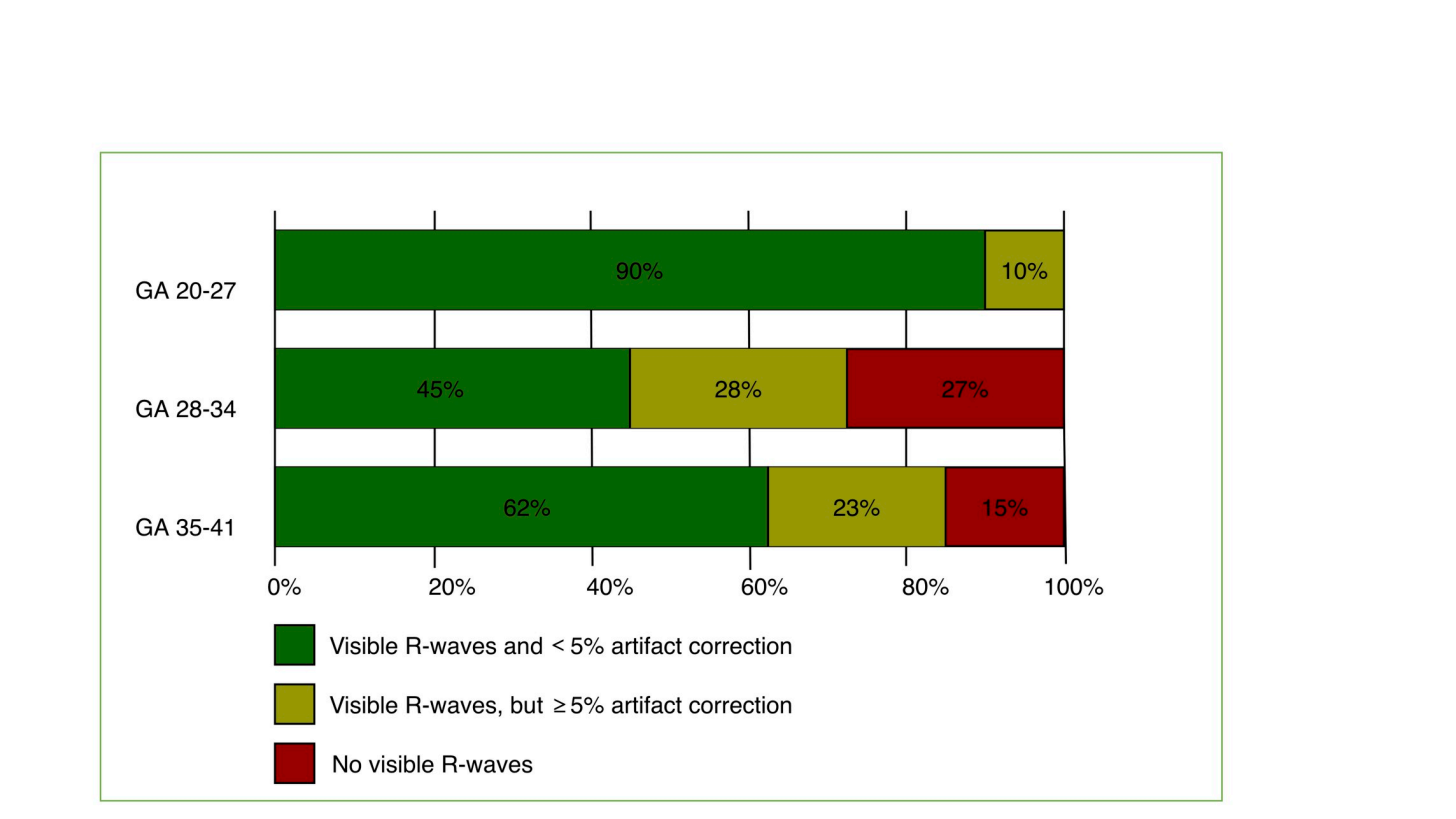
Rate of usable non-invasive fetal ECG recordings divided into gestational age (GA) in weeks.

A total of 75 healthy singleton pregnant women were recruited for this study. The mean duration of the NI-FECG was 24 minutes (range: 20–31 minutes). Of the 75 recordings, 48 (64%) fulfilled the quality criteria of at least two time series of 120 seconds with <5% artifact correction, while 56 (75%) contained at least one 120-second time series. Signal quality varied considerably with gestational age. The highest performing was the second trimester (20^+0^–27^+6^), with 90% fulfilling the quality criteria, and the lowest performing was the early third trimester (28^+0^–34^+5^), with 45% fulfilling the quality criteria (Fig 3). None of the recordings were excluded from analysis due to failure in the automatic R-wave detection.

Mean age, mean BMI, parity, smoking status, and mean GA in the three gestational age groups were comparable between women with a NI-FECG fulfilling the quality criteria (included in the reliability analyses) and all women included in the study (Table 2).

**Table 2:** Characteristics of included women.

Characteristics		All included women (n = 75)	Women included in reliability analyses (n = 48)
Maternal age (years)	Mean (95%CI)	29.5 (28.5–30.6)	28.5 (27.5–29.6)
Maternal BMI (kg/m^2^)	Mean (95%CI)	24.0 (22.9–25.1)	23.6 (22.2–25.0)
Nulliparity	n (%)	44 (60.3%)	26 (56.5%)
Smoking	n (%)	2 (2.7%)	2 (4.3%)
GA^a^ (group A)	Median (range)	23.1 (20.6–26.9)	23.1 (20.6–26.9)
GA^a^ (group B)	Median (range)	30.1 (28.3–34.9)	30.1 (28.3–34.6)
GA^a^ (group C)	Median (range)	38.1 (35.3–40.4)	38.7 (36.4–40.4)

^a^ Gestational age in weeks

### Reliability

This section examines the reliability of time domain and spectral domain analyses taking into account the impact of heart rate pattern, gestational age, and length and selection of time series on reliability.

#### Heart rate pattern

In all GA groups, more than 90% of the recordings contained SHRP. HRP I was most frequently seen in GA group A_20-27_ (52%), HRP II in GA group C_35-41_ (88%). Only HRP I and II were included in the analyses, as very few recordings (six) fulfilled the criteria of HRP III.

#### Time domain analyses (Mean RR, SDNN, RMSSD)

SHRP provided the highest ICCs and the lowest CVs in all aspects, compared to HRP. Mean RR showed very high reliability, with CVs below 1% and ICCs above 0.90 (Table 3). SDNN and RMSSD also showed high ICCs; however, CVs ranged from 20–26% (acceptable ≤ 15%) and LoA of 0.53–1.73 (in both 120- and 64-second time series; Table 3). CVs were acceptable for SDNN and RMSSD in GA group B_28-35_ being 13%-14% by a single 120 second or 64 second (only SDNN) time series (Table 4). Furthermore, the sub analyzes estimating the ICCs and CVs by using the mean of several time series showed CVs at 10–16% by the mean of two 64-second time series (S1 and S2 Tables) for SDNN and RMSSD.

**Table 3:** Reliability of time domain and spectral domain parameters from gestational age 20^+0^ to 41^+0^ divided into heart rate pattern and length of time series. (Based on the pairwise comparison of two measurements from the same recording).

**Mean RR**							
**Method**	**Length (s)**	**N**	**Median (ms)**	**Range (ms)**	**CV** ^a^	**LoA**^b^ **(ratio)**	**ICC** ^c^
SHRP	120	46	424	382–507	0.014(0.012–0.017)	0.96–1.04	0.95
SHRP	64	46	424	380–509	0.015(0.013–0.019)	0.95–1.04	0.94
HRP1	120	20	422	376–494	0.012(0.009–0.016)	0.97–1.03	0.97
HRP1	64	20	422	378–496	0.014(0.010–0.018)	0.96–1.04	0.96
HRP2	120	37	418	374–501	0.035(0.028–0.044)	0.92–1.11	0.70
HRP2	64	37	418	364–510	0.039(0.031–0.049)	0,91–1.13	0.67
**SDNN**							
**Method**	**Length (s)**	**N**	**Median (ms)**	**Range (ms)**	**CV** ^a^	**LoA**^b^ **(ratio)**	**ICC** ^c^
SHRP	120	46	6.25	1.38–28.16	0.20(0.16–0.25)	0.55–1.66	0.87
SHRP	64	46	6.30	1.31–32.98	0.26(0.21–0.32)	0.48–1.96	0.79
HRP1	120	20	5.10	1.83–12.09	0.41(0.30–0.58)	0.37–3.39	0.26
HRP1	64	20	4.83	1.51–11.88	0.44(0.32–0.63)	0.38–3.79	0.18
HRP2	120	37	8.24	1.80–22.87	0.32(0.25–0.41)	0.49–2.63	0.54
HRP2	64	37	7.56	1.74–30.72	0.41(0.32–0.53)	0.35–3.16	0.44
**RMSSD**							
**Method**	**Length (s)**	**N**	**Median (ms)**	**Range (ms)**	**CV** ^a^	**LoA**^b^ **(ratio)**	**ICC** ^c^
SHRP	120	46	4.12	0.92–17.01	0.21(0.17–0.26)	0.53–1.73	0.89
SHRP	64	46	4.10	0.99–18.81	0.25(0.20–0.31)	0.48–1.90	0.86
HRP1	120	20	3.22	0.10–7.90	0.30(0.21–0.41)	0.43–2.23	0.68
HRP1	64	20	3.14	0.93–8.75	0.32(0.23–0.44)	0.42–2.43	0.65
HRP2	120	37	4.99	1.07–16.58	0.32(0.25–0.40)	0.54–2.63	0.69
HRP2	64	37	4.54	1.13–18.67	0.37(0.29–0.48)	0.41–2.98	0.65
**LF power (FFT)**							
**Method**	**Length (s)**	**N**	**Median (ms)**	**Range (ms)**	**CV** ^a^	**LoA**^b^ **(ratio)**	**ICC** ^c^
SHRP	120	46	22.97	0.74–1317.78	0.55(0.44–0.70)	0.22–3.86	0.83
SHRP	64	46	23.25	0.75–692.27	0.77(0.60–1.00)	0.16–7.12	0.71
HRP1	120	20	15.83	1.12–175.01	1.03(0.69–1.69)	0.15–14.65	0.37
HRP1	64	20	14.60	1.60–146.25	1.53(0.96–2.90)	0.07–30.53	0.08
HRP2	120	37	37.72	0.53–575.22	1.47(1.04–2.26)	0.06–24.84	0.15
HRP2	64	37	31.28	0.75–536.83	1.35(0.97–2.04)	0.07–21.18	0.34
**HF power (FFT)**							
**Method**	**Length (s)**	**N**	**Median (ms)**	**Range (ms)**	**CV** ^a^	**LoA**^b^ **(ratio)**	**ICC** ^c^
SHRP	120	46	3.93	0.24–73.39	0.46(0.37–0.58)	0.26–2.97	0.89
SHRP	64	46	3.91	0.27–137.45	0.67(0.53–0.86)	0.17–5.11	0.82
HRP1	120	20	2.31	0.29–18.36	0.63(0.44–0.93)	0.19–5.21	0.74
HRP1	64	20	2.09	0.19–21.19	0.78(0.54–1.18)	0.20–8.51	0.65
HRP2	120	37	5.23	0.24–96.32	0.95(0.71–1.33)	0.24–12.45	0.62
HRP2	64	37	4.25	0.21–89.88	1.03(0.77–1.47)	0.10–11.87	0.60
**LF/HF (FFT)**							
**Method**	**Length (s)**	**N**	**Median (ms)**	**Range (ms)**	**CV** ^a^	**LoA**^b^ **(ratio)**	**ICC** ^c^
SHRP	120	46	5.84	0.41–92.61	0.64(0.51–0.82)	0.20–5.34	0.71
SHRP	64	46	5.95	0.40–52.75	0.99(0.76–1.35)	0.11–11.45	0.51
HRP1	120	20	6.86	1.17–59.08	0.78(0.54–1.19)	0.25–8.96	0.47
HRP1	64	20	6.99	1.19–85.08	0.87(0.60–1.36)	0.14–9.57	0.40
HRP2	120	37	7.21	0.39–408.84	1.57(1.10–2.48)	0.03–16.05	0.19
HRP2	64	37	7.36	0.51–101.84	1.20(0.87–1.76)	0.08–16.15	0.30
	**LF(AR)**						
**Method**	**Length (s)**	**N**	**Median (ms)**	**Range (ms)**	**CV** ^a^	**LoA**^b^ **(ratio)**	**ICC** ^c^
SHRP	120	46	24.03	1.10–653.58	0.48(0.38–0.60)	0.26–3.18	0.85
SHRP	64	46	2304	0.88–613.24	0.64(0.51–0.83)	0.19–5.16	0.76
HRP1	120	20	16.77	1.71–109.09	1.13(0.75–1.90)	0.11–16.46	0.15
HRP1	64	20	14.70	0.97–119.45	1.28(0.83–2.26)	0.11–22.38	0.13
HRP2	120	37	44.87	1.17–364.50	0.78(0.59–1.05)	0.21–8.40	0.54
HRP2	64	37	36.40	0.69–3127.55	1.38(0.98–2.10)	0.06–20.77	0.29
	**HF(AR)**						
**Method**	**Length (s)**	**N**	**Median (ms)**	**Range (ms)**	**CV** ^a^	**LoA**^b^ **(ratio)**	**ICC** ^c^
SHRP	120	46	3.98	0.23–71.04	0.46(0.37–0.58)	0.27–3.09	0.88
SHRP	64	46	3.71	0.24–90.18	0.54(0.43–0.68)	0.22–3.45	0.86
HRP1	120	20	2.44	0.26–16.79	0.63(0.44–0.93)	0.19–5.15	0.71
HRP1	64	20	2.19	0.20–21.25	0.67(0.47–1.00)	0.21–6.35	0.70
HRP2	120	37	5.55	0.24–66.40	0.74(0.56–0.99)	0.25–7.87	0.70
HRP2	64	37	4.32	0.24–81.98	0.78(0.60–1.06)	0.17–8.01	0.70
	**LF/HF (AR)**						
**Method**	**Length (s)**	**N**	**Median (ms)**	**Range (ms)**	**CV** ^a^	**LoA**^b^ **(ratio)**	**ICC** ^c^
SHRP	120	46	6.03	0.40–43.91	0.46(0.37–0.58)	0.29–3.39	0.81
SHRP	64	46	6.21	0.37–67.94	0.67(0.53–0.87)	0.21–6.23	0.71
HRP1	120	20	6.88	0.97–46.54	0.77(0.53–1.17)	0.21–8.52	0.39
HRP1	64	20	6.72	1.25–79.56	0.83(0.57–1.29)	0.19–9.84	0.49
HRP2	120	37	8.08	0.55–199.14	0.86(0.65–1.18)	0.12–7.61	0.53
HRP2	64	37	8.43	0.59–1213.93	1.36(0.97–2.04)	0.06–17.30	0.33

^a^ Within Coefficient of Variation (CV).

^b^ Within 95% Limits of Agreement (LoA) between measurement A and B in the same recording.

^c^ Intraclass Correlation Coefficient (ICC).

**Table 4:** Reliability of SDNN and RMSSD divided into gestational age groups, heart rate pattern and length of time series. (Based on the pairwise comparison of two measurements from the same recording).

**Gestational age group A: 20** ^**+0**^ **–27** ^**+6**^							
**SDNN**							
**Method**	**Length (s)**	**n**	**Median (ms)**	**Range (ms)**	**CV** ^a^	**LoA**^b^ **(ratio)**	**ICC** ^c^
SHRP	120	18	4.59	1.38–9.06	0.22(0.16–0.31)	0.52–1.72	0.77
SHRP	64	18	4.70	1.31–8.84	0.27(0.20–0.39)	0.45–2.07	0.63
HRP1	120	10	4.16	1.83–11.75	0.37(0.23–0.60)	0.32–2.50	0.43
HRP1	64	10	3.84	1.51–8.56	0.42(0.26–0.69)	0.33–3.41	0.23
HRP2	120	14	6.77	1.80–22.87	0.45(0.30–0.68)	0.43–3.91	0.44
HRP2	64	14	5.83	1.74–30.72	0.58(0.39–0.92)	0.23–5.02	0.27
**RMSSD**							
**Method**	**Length (s)**	**N**	**Median (ms)**	**Range (ms)**	**CV** ^a^	**LoA**^b^ **(ratio)**	**ICC** ^c^
SHRP	120	18	2.50	0.92–7.48	0.28(0.20–0.40)	0.42–1.89	0.69
SHRP	64	18	2.49	0.99–7.01	0.29(0.21–0.42)	0.40–1.92	0.69
HRP1	120	10	2.40	1.00–7.18	0.33(0.21–0.53)	0.35–1.97	0.59
HRP1	64	10	2.35	0.93–8.75	0.33(0.21–0.53)	0.39–2.57	0.61
HRP2	120	14	3.41	1.07–7.67	0.39(0.26–0.58)	0.50–3.31	0.51
HRP2	64	14	2.83	1.13–8.53	0.47(0.31–0.72)	0.33–4.02	0.35
Gestational age group B: 28^+0^–34^+6^							
SDNN							
**Method**	**Length (s)**	**N**	**Median (ms)**	**Range (ms)**	**CV** ^a^	**LoA**^b^ **(ratio)**	**ICC** ^c^
SHRP	120	12	8.68	3.06–22.13	0.13(0.09–0.20)	0.66–1.39	0.93
SHRP	64	12	8.95	3.19–25.56	0.14(0.10–0.21)	0.65–1.47	0.93
HRP2	120	9	8.83	5.74–22.10	0.16(0.10–0.25)	0.70–1.66	0.78
HRP2	64	9	8.29	3.96–22.12	0.22(0.14–0.36)	0.63–2.05	0.68
RMSSD							
**Method**	**Length (s)**	**N**	**Median (ms)**	**Range (ms)**	**CV** ^a^	**LoA**^b^ **(ratio)**	**ICC** ^c^
SHRP	120	12	5.37	2.13–17.01	0.13(0.09–0.20)	0.73–1.51	0.94
SHRP	64	12	5.28	2.09–18.81	0.23(0.15–0.35)	0.55–2.00	0.84
HRP2	120	9	5.53	2.83–16.58	0.29(0.18–0.47)	0.54–2.51	0.55
HRP2	64	9	5.41	2.48–18.67	0.36(0.22–0.60)	0.36–2.80	0.46
**Gestational age group C: 35** ^**+0**^ **–41** ^**+0**^							
SDNN							
**Method**	**Length (s)**	**N**	**Median (ms)**	**Range (ms)**	**CV** ^a^	**LoA**^b^ **(ratio)**	**ICC** ^c^
SHRP	120	16	6.90	3.30–28.16	0.23(0.16–0.32)	0.51–1.83	0.83
SHRP	64	16	6.72	3.39–32.98	0.30(0.21–0.44)	0.42–2.25	0.69
HRP2	120	14	9.61	5.62–21.48	0.25(0.17–0.37)	0.50–2.08	0.49
HRP2	64	14	9.24	4.53–23.11	0.30(0.21–0.45)	0.42–2.28	0.37
RMSSD							
**Method**	**Length (s)**	**n**	**Median (ms)**	**Range (ms)**	**CV** ^a^	**LoA**^b^ **(ratio)**	**ICC** ^c^
SHRP	120	16	5.90	2.76–13.13	0.17(0.12–0.25)	0.60–1.61	0.87
SHRP	64	16	5.93	2.94–14.71	0.21(0.15–0.30)	0.54–1.77	0.81
HRP2	120	14	6.82	3.01–14.31	0.25(0.17–0.36)	0.60–2.18	0.66
HRP2	64	14	6.51	2.50–12.83	0.26(0.18–0.38)	0.58–2.26	0.63

^a^ Within Coefficient of Variation (CV)

^b^ Within 95% Limits of Agreement (LoA) between measurement A and B in the same recording

^c^ Intraclass Correlation Coefficient (ICC)

For GA group C_35-41_, the CVs were 12–16% by the mean of two, 120-second time series and 12–17% by the mean of three, 64 -second time series. In GA group A_20-27_ the mean of four time series (120 and 64 seconds) was needed to achieve low CVs between 11 and 14%.

Generally, HRP II needed more time series than SHRV to achieve acceptable reliability, and in GA group A_20-27_ acceptable reliability was almost never achieved.

Due to few observations in HPR I in GA group B_28-35_, no reliability analyses were performed, but acceptable reliability was achieved for RMSSD in GA group A_20-27_ in the analyses estimating reliability by the mean of at least five measurements (S2 Table).

### Spectral domain analyses (LF-power, HF-power, LF/HF-power)

Outliers were seen in spectral analyses that were as high as ten times the median value. These outliers were interpreted as true and kept in the analyses, as the accuracy of these measurements were systematically checked via validation of the FECG quality, R-wave detections, and artifact correction. No artifacts (ectopic or missing beat) or incorrect detection of R-waves were identified in these outliers.

In SHRP, relative reliability was high, with ICCs > 0.80 shown for both LF-power (FFT+AR) and HF-power (FFT+AR) in 120-seconds time series (Table 3). However, the absolute reliability was low (CV: 46–67%), and the narrowest LoA was 0.26 to 2.97.

However, in the sub analyses estimating reliability by the mean of more than one measurement, HF-power (AR+FFT) achieved CVs at 14% by the mean of four, 120-second time series in GA group B_28-35_ (S3 and S4 Tables). Length of time series did affect reliability, however, as for time domain analyses, stationarity and GA were more important factors.

LF-power (FFT+AR) and LF/HF failed to achieve acceptable reliability except from LF(AR) in GA group B_28-34_ (S5 Table).

## Discussion

### Main findings

Time domain parameters achieved acceptable reliability in GA group B_28-35_, while spectral analyses showed high CVs, but also high ICCs. However, sub analyses indicate that by using the mean of two to six time series, acceptable reliability is achievable for time domain analyses in all GA groups and for HF power (FFT and AR) from GA 28^+0^ and LF power (AR) in GA group B_28-35_. Stationarity of mean RR (SHRP) seemed important, and the highest reliability was found in SHRP.

### Strengths and limitations

We minimized the risk of information bias by an accurate detection of RR-intervals (milliseconds of accuracy) and by carefully processing the data both visually and technically, from filtering raw data to artifact correction and replacement by Cubic Spline. As fetal R-wave detection obviously is more challenging than R-wave detection in adults, it is a strength that every time series in this study was systematically checked for errors in the fetal R-wave detection, and only recordings containing visible fetal R-waves were included in the analyses.

In order to diminish detection bias, categorization of time series into the predefined heart rate pattern was done by at least two authors, blinded to the other assessor’s evaluation. Furthermore, outcomes were blinded to the assessor during the selection of time series and performance of FHRV analyses.

Maternal position has been shown to affect both time domain and spectral domain parameters [30], so shifting maternal position during acquisition of NI-FECG might bias our results. However, the same maternal position was maintained throughout most recordings.

The study was not powered to evaluate the effect of fetal sex [17, 18], maternal smoking [15, 16] or ethnicity [19]. Furthermore, fetal movements were not detected or addressed [28, 31].

### Interpretation

High quality NI-FECG has been reported to be very difficult to achieve between 28 to 34 gestational weeks [28]. In this study we reached a success rate (45%) of good quality NI-FECG with less than 5% artifact correction (in at least two two-minute periods). In addition, fetal R-waves were intermittently visualized in another 28%, adding optimism about this clinically feasible method. However, to achieve an increased success rate, it may be necessary to include reduction of noise from surroundings and individual electrode placement depending on fetal position. However, the most challenging factor might be the vernix caseosa [39], but electrode placement based on the thickness of this layer [40] and optimization of the algorithm for R-wave detection [41] have the potential to increase the success rate of R-wave detection in NI-FECG. Allowing more artifact corrections would also lead to a higher rate of usable NI-FECG in these specific weeks. However, we chose a low correction rate of 5%, as we wanted to minimize the risk of information bias.

However, not only a feasible method, but also reliable measurements are needed for surveillance. Previous studies investigating the reliability of FHRV have not been identified. The results from this present study showed high relative reliability, indicated by excellent ICCs in most time and spectral domain parameters. This means that FHRV measurements mainly reflect the “true value” relative to random error (within fetus variance). Nevertheless, absolute reliability, based on CV and LoA, revealed the existence of substantial random error, especially in spectral analyses. In the scope of fetal monitoring, high absolute reliability is important (low CV and narrow LoA). Otherwise, variations in FHRV due to fetal compromise would be hidden in the within variation. By using a few measurements, the challenges of low absolute reliability were overcome in time domain parameters, especially after gestational week 28. Earlier gestational weeks and spectral analyses required more measurements; thus, conclusions are more uncertain in this perspective. Furthermore, LF power may have been more reliable if longer time series were used, as the lower frequencies of the LF band may be underestimated in our 64-second time series.

The presence of outliers and the difference in mean RR were rejected to be the main reason for the high absolute reliability (CVs) found in this study. Thus, reliability increased only slightly when statistics were applied without outliers, and the median difference in mean RR within the fetus was less than 1%.

The tendencies of high relative reliability (ICC) and low absolute reliability (CV) have also been shown in healthy adults [38]. In fetuses, this might be a sign of a well-developed autonomic nervous system, which is able to change with shifting fetal state. Restricting the analyses to a certain fetal heart rate pattern may to a certain extent compensate for the uncontrolled fetal movements. However, fetal movements may occur in each state, and the strong association between HRP and movements is only found after 35 weeks of gestation [11]. Furthermore, breathing movements are intermittent in all HRP [42]. Therefore, considering fetal movements in the process of selecting time series would probably improve reliability. Furthermore, a combination of stationarity and HRP would probably also increase reliability.

We found acceptable reliability for MeanRR, SDNN and RMSSD, when SHRP was fulfilled in selected time series. Sub analyses indicate that the mean value of more measurements increase reliability and acceptable reliability also for spectral parameters. However, these analyses rely on the assumption, that the variance is the same in additional selected time series. Therefore, these analyses need further evaluation using longer recordings and more measurements.

### Clinical applicability

We consider FHRV based on NI-FECG as clinically applicable. The NI-FECG recording is very easy to perform and may even be performed by the pregnant women herself in a home-management setting [43]. In hospitals not offering home-management the NI-FECG recording can be performed in the outpatient clinic by most healthcare professionals. Furthermore, the selection of time series and calculation of FHRV can be automated by algorithms. The equipment is cheap compared to, for example, Doppler ultrasound, which is also used as a diagnostic tool. Most importantly, FHRV has shown interesting associations with fetal compromise both in pregnancy and during labor [7, 9, 44, 45]. However, there remains some challenges that need to be solved before the method can be clinically implementable, including a higher performance of NI-FECG between GA week 28 and 34, which is discussed above, and automatic selection of appropriate time series, which probably could be solved by an algorithm based on the findings in this study and future studies.

## Conclusion

NI-FECG represents a valuable alternative to CTG, as it covers both accurate FHRV analysis, including time domain and spectral domain analysis, and the classic CTG interpretation. We showed that the method is feasible, has acceptable reliability for SDNN, RMSSD, and HF-power was achievable. Time domain parameters generally showed higher reliability than spectral domain parameters, and GA from 28^+0^ weeks performed better than earlier gestational ages.

Future studies need to focus on the importance of other factors possibly affecting FHRV, including fetal body and breathing movements. Furthermore, day to day reliability also needs to be evaluated. Transparent studies based on proper signal acquisition, processing and optimal artifact correction are prerequisites for reliable FHRV investigations.

## Supporting information

S1 Table Reliability of SDNN within the fetus as a function of average of n measurements. (Divided by gestational age). ^a^ Gestational age weeks. ^b^ 95% Prediction interval within fetus as compared to the true median level as a function of average of n measurements. ^c^ Coefficient of variation. ^d^ Intraclass correlation coefficient.
(PDF)

S2 Table Reliability of RMSSD within the fetus as a function of average of n measurements. (Divided by gestational age). a Gestational age in weeks. ^b^ 95% Prediction interval within fetus as compared to the true median level as a function of average of n measurements. ^c^ Coefficient of variation. ^d^ Intraclass correlation coefficient.
(PDF)

S3 Table Reliability of HF-power (FFT) within the fetus as a function of average of n measurements. Divided by gestational age. † Gestational age weeks. ‡ 95% Prediction interval within fetus as compared to the true median level as a function of average of n measurements. § Coefficient of variation. ⨎Intraclass correlation coefficient.
(PDF)

S4 Table Reliability of HF-power (AR) within the fetus as a function of average of n measurements. Divided by gestational age. ^a^ Gestational age weeks. ^b^ 95% Prediction interval within fetus as compared to the true median level as a function of average of n measurements. ^c^ Coefficient of variation. ^d^ Intraclass correlation coefficient.
(PDF)

S5 Table Reliability of LF-power (AR) within the fetus as a function of average of n measurements. Divided by gestational age. ^a^ Gestational age weeks. ^b^ 95% Prediction interval within fetus as compared to the true median level as a function of average of n measurements. ^c^ Coefficient of variation. ^d^ Intraclass correlation coefficient.
(PDF)

S1 Data (DTA)

## Abbreviations

AR: Autoregressive model
bpm: Beats per minute
CRL: Crown rump length
CTG: Cardiotocography
CV: Coefficient of variance
FBS: Fetal behavioral states
FFT: Fast Fourier Transformation
FHRV: Fetal heart rate variability
GA: Gestational age
HF: High frequency
HRP: Heart rate pattern
HRV: Heart rate variability
ICC: Intraclass correlation coefficient
LF: Low frequency
LoA: Limits of agreement
NI-FECG: Noninvasive fetal electrocardiography
RMSSD: Root mean square of successive RR-interval differences
RR-interval: Time interval between successive ECG R-waves
SDNN: Standard deviation of normal to normal RR-intervals
SHRP: Stationary heart rate pattern
