# Supplementary material for: Towards better reliability in fetal heart rate variability using time domain and spectral domain analyses. A new method for assessing fetal neurological state?
Source: PLoS One. 2022 Mar 1;17(3):e0263272. doi: 10.1371/journal.pone.0263272 (PMC8887753; doi:10.1371/journal.pone.0263272)
Supplement: S1 Table — (Divided by gestational age). a Gestational age weeks. b 95% Prediction interval within fetus as compared to the true median level as a function of average of n measurements. c Coefficient of variation. d Intraclass correlation coefficient. (PDF) [file pone.0263272.s001.pdf]

GA[a] 20-27					GA[a] 28-34					GA [a]35-41				
n	Within PI[b] (ratio)		Within CV[c]	ICC[d]	n	Within PI[b] (ratio)		Within CV[c]	ICC[d]	n	Within PI[b] (ratio)		Within CV[c]	ICC[d]
SHRP 120 s					AZ 120 s					AZ 120 s				
1	0.66	1.53	0.22	0.77	1	0.77	1.30	**0.13**	**0.93**	1	0.65	1.55	0.23	**0.83**
2	0.74	1.35	0.15	**0.87**	2	0.83	1.20	**0.09**	**0.96**	2	0.73	1.36	0.16	**0.91**
3	0.78	1.28	**0.12**	**0.93**	3	0.86	1.16	**0.08**	**0.97**	3	0.78	1.29	**0.13**	**0.93**
4	0.81	1.24	**0.11**	**0.93**	4	0.88	1.14	**0.07**	**0.98**	4	0.80	1.24	**0.11**	**0.95**
5	0.83	1.21	**0.10**	**0.94**	5	0.89	1.12	**0.06**	**0.98**	5	0.82	1.22	**0.10**	**0.96**
6	0.84	1.19	**0.09**	**0.95**	6	0.90	1.11	**0.05**	**0.99**	6	0.84	1.19	**0.09**	0.97
SHRP 64 s					SHRP 64 s					SHRP 64 s				
1	0.59	1.70	0.27	0.63	1	0.76	1.32	**0.14**	**0.93**	1	0.56	1.78	0.30	0.69
2	0.69	1.45	0.19	0.77	2	0.82	1.22	**0.10**	**0.96**	2	0.66	1.50	0.21	**0.81**
3	0.74	1.36	0.16	**0.84**	3	0.85	1.17	**0.08**	**0.97**	3	0.72	1.40	0.17	**0.87**
4	0.77	1.30	**0.14**	**0.87**	4	0.87	1.15	**0.07**	**0.98**	4	0.75	1.33	**0.15**	**0.90**
5	0.79	1.27	**0.12**	**0.89**	5	0.88	1.13	**0.06**	**0.98**	5	0.77	1.29	**0.13**	**0.92**
6	0.81	1.24	**0.11**	**0.91**	6	0.89	1.12	**0.06**	**0.99**	6	0.79	1.27	**0.12**	**0.93**
HRP1 120 s					HRP1 120 s					HRP1 120 s				
1	0.50	2.02	0.37	0.43	few observations					few observations				
2	0.61	1.64	0.26	0.60										
3	0.67	1.50	0.21	0.70										
4	0.70	1.42	0.18	0.75										
5	0.73	1.37	0.16	0.79										
6	0.75	1.33	0.15	**0.82**										
HRP1 64 s					HRP1 64 s					HRP1 64 s				
1	0.45	2.20	0.42	0.23	few observations					few observations				
2	0.57	1.75	0.29	0.38										
3	0.63	1.58	0.24	0.48										
4	0.67	1.48	0.20	0.55										
5	0.70	1.42	0.18	0.60										
6	0.72	1.38	0.17	0.64										
HRP2 120s					HRP2 120s					HRP2 120s				
1	0.43	2.30	0.45	0.44	1	0.74	1.36	0.16	0.78	1	0.62	1.62	0.25	0.49
2	0.55	1.80	0.31	0.61	2	0.80	1.24	**0.11**	**0.87**	2	0.71	1.41	0.18	0.66

3	0.62	1.62	0.25	0.70	3	0.84	1.19	**0.09**	**0.91**	3	0.76	1.32	**0.14**	0.74
4	0.66	1.52	0.22	0.76	4	0.86	1.17	**0.08**	**0.93**	4	0.78	1.27	**0.12**	0.79
5	0.69	1.45	0.19	**0.80**	5	0.87	1.15	**0.07**	**0.95**	5	0.80	1.24	**0.11**	**0.83**
6	0.71	1.41	0.17	**0.82**	6	0.88	1.13	**0.06**	**0.95**	6	0.82	1.22	**0.10**	**0.85**
HRP2 64 s					HRP2 64 s					HRP2 64 s				
1	0.35	2.88	0.58	0.27	1	0.65	1.54	0.22	0.68	1	0.56	1.78	0.30	0.37
2	0.47	2.11	0.40	0.42	2	0.74	1.36	0.16	**0.81**	2	0.66	1.50	0.21	0.54
3	0.54	1.84	0.32	0.52	3	0.78	1.28	**0.13**	**0.86**	3	0.72	1.40	0.17	0.63
4	0.59	1.70	0.28	0.60	4	0.81	1.24	**0.11**	**0.89**	4	0.75	1.33	**0.15**	0.70
5	0.62	1.61	0.25	0.65	5	0.82	1.21	**0.10**	**0.91**	5	0.77	1.29	**0.13**	0.74
6	0.65	1.54	0.22	0.69	6	0.84	1.19	**0.09**	**0.93**	6	0.79	1.27	**0.12**	0.78
