# Supplementary material for: Towards better reliability in fetal heart rate variability using time domain and spectral domain analyses. A new method for assessing fetal neurological state?
Source: PLoS One. 2022 Mar 1;17(3):e0263272. doi: 10.1371/journal.pone.0263272 (PMC8887753; doi:10.1371/journal.pone.0263272)
Supplement: S2 Table — (Divided by gestational age). a Gestational age in weeks. b 95% Prediction interval within fetus as compared to the true median level as a function of average of n measurements. c Coefficient of variation. d Intraclass correlation coefficient. (PDF) [file pone.0263272.s002.pdf]

GA[a] 20-27					GA[a] 28-34					GA [a]35-41				
**n**	**Within PI[b] (ratio)**		**Within CV[c]**	**ICC[d]**	**n**	**Within PI[b] (ratio)**		**Within CV[c]**	**ICC[d]**	**n**	**Within PI[b] (ratio)**		**Within CV[c]**	**ICC[d]**
SHRP 120 s					SHRP 120 s					SHRP 120 s				
1	0.58	1.72	0.28	0.69	1	0.77	1.29	**0.13**	**0.94**	1	0.71	1.40	0.17	**0.87**
2	0.68	1.47	0.20	**0.81**	2	0.83	1.20	**0.09**	**0.97**	2	0.79	1.27	**0.12**	**0.93**
3	0.73	1.37	0.16	**0.87**	3	0.86	1.16	**0.08**	**0.98**	3	0.82	1.21	**0.10**	**0.95**
4	0.76	1.31	**0.14**	**0.90**	4	0.88	1.14	**0.07**	**0.98**	4	0.84	1.18	**0.09**	**0.96**
5	0.78	1.27	**0.12**	**0.92**	5	0.89	1.12	**0.06**	**0.99**	5	0.86	1.16	**0.08**	**0.97**
6	0.80	1.25	**0.11**	**0.93**	6	0.90	1.11	**0.05**	**0.99**	6	0.87	1.15	**0.07**	**0.98**
SHRP 64 s					SHRP 64 s					SHRP 64 s				
1	0.57	1.76	0.29	0.69	1	0.64	1.55	0.23	**0.84**	1	0.66	1.51	0.21	**0.81**
2	0.67	1.49	0.21	**0.81**	2	0.73	1.37	0.16	**0.92**	2	0.75	1.34	**0.15**	**0.90**
3	0.72	1.39	0.17	**0.87**	3	0.78	1.29	**0.13**	**0.94**	3	0.79	1.27	**0.12**	**0.93**
4	0.75	1.33	**0.14**	**0.90**	4	0.80	1.25	**0.11**	**0.96**	4	0.81	1.23	**0.11**	**0.95**
5	0.78	1.29	**0.13**	**0.92**	5	0.82	1.22	**0.10**	**0.96**	5	0.83	1.20	**0.09**	**0.96**
6	0.79	1.26	**0.12**	**0.93**	6	0.84	1.20	**0.09**	**0.97**	6	0.85	1.18	**0.09**	**0.96**
HRP1 120 s					HRP1 120 s					HRP1 120 s				
1	0.53	1.88	0.33	0.59	few observations					few observations				
2	0.64	1.56	0.23	0.74										
3	0.70	1.44	0.19	**0.81**										
4	0.73	1.37	0.16	**0.85**										
5	0.75	1.32	**0.14**	**0.88**										
6	0.77	1.29	**0.13**	**0.90**										
HRP1 64 s					HRP1 64 s					HRP1 64 s				
1	0.53	1.89	0.33	0.61	few observations					few observations				
2	0.64	1.57	0.23	0.76										
3	0.69	1.44	0.19	**0.82**										
4	0.73	1.37	0.16	**0.86**										
5	0.75	1.33	**0.15**	**0.89**										
6	0.77	1.30	**0.13**	**0.90**										
HRP2 120s					HRP2 120s					HRP2 120s				
1	0.53	1.89	0.33	0.51	1	0.57	1.74	0.29	0.55	1	0.62	1.61	0.25	0.66
2	0.64	1.57	0.23	0.68	2	0.68	1.48	0.20	0.71	2	0.71	1.40	0.17	0.79

3	0.69	1.44	0.19	0.76	3	0.73	1.38	0.16	0.79	3	0.76	1.32	**0.14**	**0.85**
4	0.73	1.37	0.16	**0.81**	4	0.76	1.32	**0.14**	**0.83**	4	0.79	1.27	**0.12**	**0.89**
5	0.75	1.33	**0.15**	**0.84**	5	0.78	1.28	**0.13**	**0.86**	5	0.81	1.24	**0.11**	**0.91**
6	0.77	1.30	**0.13**	**0.86**	6	0.80	1.25	**0.12**	0.88	6	0.82	1.22	**0.10**	**0.92**
HRP2 64 s					HRP2 64 s					HRP2 64 s				
1	0.42	2.39	0.47	0.35	1	0.50	1.98	0.36	0.46	1	0.61	1.65	0.26	0.63
2	0.54	1.85	0.32	0.52	2	0.62	1.62	0.25	0.63	2	0.70	1.42	0.18	0.78
3	0.60	1.65	0.26	0.62	3	0.67	1.48	0.20	0.72	3	0.75	1.33	**0.15**	**0.84**
4	0.65	1.55	0.23	0.68	4	0.71	1.41	0.18	0.77	4	0.78	1.28	**0.13**	**0.87**
5	0.68	1.48	0.20	0.73	5	0.74	1.36	0.16	**0.81**	5	0.80	1.25	**0.11**	**0.90**
6	0.70	1.43	0.18	0.76	6	0.76	1.32	**0.14**	**0.84**	6	0.82	1.23	**0.10**	**0.91**
