# Supplementary material for: Towards better reliability in fetal heart rate variability using time domain and spectral domain analyses. A new method for assessing fetal neurological state?
Source: PLoS One. 2022 Mar 1;17(3):e0263272. doi: 10.1371/journal.pone.0263272 (PMC8887753; doi:10.1371/journal.pone.0263272)
Supplement: S3 Table — Divided by gestational age. † Gestational age weeks. ‡ 95% Prediction interval within fetus as compared to the true median level as a function of average of n measurements. § Coefficient of variation. ⨎Intraclass correlation coefficient. (PDF) [file pone.0263272.s003.pdf]

GA† 20-27					GA† 28-34					GA †35-41				
**n**	**Within PI‡ (ratio)**		**Within CV§**	**ICC∱**	**n**	**Within PI‡ (ratio)**		**Within CV§**	**ICC∱**	**n**	**Within PI‡ (ratio)**		**Within CV§**	**ICC∱**
AZ 120 s					AZ 120 s					AZ 120 s				
1	0.33	3.06	0.62	0.71	1	0.58	1.74	0.29	**0.94**	1	0.49	2.03	0.37	**0.86**
2	0.45	2.21	0.42	**0.83**	2	0.68	1.48	0.20	**0.97**	2	0.61	1.65	0.26	**0.92**
3	0.52	1.91	0.34	**0.91**	3	0.73	1.38	0.16	**0.99**	3	0.66	1.51	0.21	**0.96**
4	0.57	1.75	0.29	**0.91**	4	0.76	1.32	**0.14**	**0.99**	4	0.70	1.42	0.18	**0.96**
5	0.61	1.65	0.26	**0.93**	5	0.78	1.28	**0.13**	**0.99**	5	0.73	1.37	0.16	**0.97**
6	0.63	1.58	0.24	**0.94**	6	0.80	1.25	**0.12**	**0.99**	6	0.75	1.34	**0.15**	**0.97**
AZ 64 s					AZ 64 s					AZ 64 s				
1	0.24	4.22	0.85	0.61	1	0.27	3.72	0.75	0.77	1	0.52	1.94	0.35	**0.87**
2	0.36	2.77	0.56	0.76	2	0.40	2.53	0.50	**0.87**	2	0.63	1.60	0.24	**0.93**
3	0.44	2.30	0.44	**0.82**	3	0.47	2.13	0.40	**0.91**	3	0.68	1.47	0.20	**0.95**
4	0.49	2.06	0.38	**0.86**	4	0.52	1.93	0.34	**0.93**	4	0.72	1.39	0.17	**0.96**
5	0.53	1.90	0.34	**0.89**	5	0.56	1.80	0.31	**0.94**	5	0.74	1.35	**0.15**	**0.97**
6	0.56	1.80	0.31	**0.90**	6	0.59	1.71	0.28	**0.95**	6	0.76	1.31	**0.14**	**0.98**
HRP1 120 s					HRP1 120 s					HRP1 120 s				
1	0.39	2.56	0.51	0.76	few observations					few observations				
2	0.51	1.95	0.35	**0.86**										
3	0.58	1.72	0.28	**0.90**										
4	0.62	1.60	0.24	**0.93**										
5	0.66	1.52	0.22	**0.94**										
6	0.68	1.47	0.20	**0.95**										
HRP1 64 s					HRP1 64 s					HRP1 64 s				
1	0.23	4.34	0.87	0.61	few observations					few observations				
2	0.35	2.82	0.57	0.76										
3	0.43	2.33	0.45	**0.82**										
4	0.48	2.08	0.39	**0.86**										
5	0.52	1.93	0.34	**0.89**										
6	0.55	1.82	0.31	**0.90**										
HRP2 120s					HRP2 120s					HRP2 120s				
1	0.22	4.48	0.89	0.51	1	0.15	6.70	1.25	0.00	1	0.27	3.68	0.75	0.60
2	0.35	2.89	0.58	0.68	2	0.26	3.84	0.78	0.00	2	0.40	2.51	0.50	0.75

3	0.42	2.38	0.46	0.76	3	0.33	3.00	0.61	0.00	3	0.47	2.12	0.40	**0.82**
4	0.47	2.12	0.40	**0.81**	4	0.39	2.59	0.52	0.00	4	0.52	1.92	0.34	**0.86**
5	0.51	1.96	0.35	**0.84**	5	0.43	2.34	0.46	0.00	5	0.56	1.79	0.30	**0.88**
6	0.54	1.85	0.32	**0.86**	6	0.46	2.17	0.41	0.00	6	0.59	1.70	0.28	**0.90**
HRP2 64 s					HRP2 64 s					HRP2 64 s				
1	0.15	6.59	1.23	0.31	1	0.16	6.41	1.21	0.21	1	0.28	3.56	0.72	0.59
2	0.26	3.79	0.77	0.48	2	0.27	3.72	0.75	0.34	2	0.41	2.46	0.48	0.74
3	0.34	2.97	0.60	0.58	3	0.34	2.92	0.59	0.44	3	0.48	2.08	0.39	**0.81**
4	0.39	2.57	0.51	0.65	4	0.39	2.53	0.50	0.51	4	0.53	1.89	0.33	**0.85**
5	0.43	2.32	0.45	0.70	5	0.44	2.30	0.44	0.56	5	0.57	1.76	0.30	**0.88**
6	0.46	2.16	0.41	0.73	6	0.47	2.14	0.40	0.61	6	0.60	1.68	0.27	**0.90**
