# Supplementary material for: Towards better reliability in fetal heart rate variability using time domain and spectral domain analyses. A new method for assessing fetal neurological state?
Source: PLoS One. 2022 Mar 1;17(3):e0263272. doi: 10.1371/journal.pone.0263272 (PMC8887753; doi:10.1371/journal.pone.0263272)
Supplement: S4 Table — Divided by gestational age. a Gestational age weeks. b 95% Prediction interval within fetus as compared to the true median level as a function of average of n measurements. c Coefficient of variation. d Intraclass correlation coefficient. (PDF) [file pone.0263272.s004.pdf]

GA[a] 20-27					GA[a] 28-34					GA [a]35-41				
**n**	**Within PI[b] (ratio)**		**Within CV[c]**	**ICC[d]**	**n**	**Within PI[b] (ratio)**		**Within CV[c]**	**ICC[d]**	**n**	**Within PI[b] (ratio)**		**Within CV[c]**	**ICC[d]**
SHRP 120 s					SHRP 120 s					SHRP 120 s				
1	0.32	3.16	0.64	0.66	1	0.58	1.72	0.28	**0.94**	1	0.51	1.95	0.35	**0.87**
2	0.44	2.25	0.43	0.80	2	0.68	1.47	0.20	**0.97**	2	0.62	1.61	0.25	**0.93**
3	0.51	1.94	0.35	0.89	3	0.73	1.37	0.16	**0.98**	3	0.68	1.47	0.20	**0.96**
4	0.56	1.78	0.30	0.89	4	0.76	1.31	**0.14**	**0.98**	4	0.72	1.40	0.17	**0.96**
5	0.60	1.67	0.27	0.91	5	0.78	1.27	**0.12**	**0.99**	5	0.74	1.35	0.15	**0.97**
6	0.63	1.60	0.24	0.92	6	0.80	1.25	**0.11**	**0.99**	6	0.76	1.31	**0.14**	**0.98**
SHRP 64 s					SHRP 64 s					SHRP 64 s				
1	0.29	3.40	0.69	0.66	1	0.41	2.44	0.48	**0.86**	1	0.48	2.09	0.39	**0.84**
2	0.42	2.38	0.46	0.80	2	0.53	1.88	0.33	**0.93**	2	0.59	1.68	0.27	**0.91**
3	0.49	2.03	0.37	0.85	3	0.60	1.67	0.27	**0.95**	3	0.65	1.53	0.22	**0.94**
4	0.54	1.84	0.32	0.89	4	0.64	1.56	0.23	**0.96**	4	0.69	1.44	0.19	**0.95**
5	0.58	1.73	0.28	0.91	5	0.67	1.49	0.21	**0.97**	5	0.72	1.39	0.17	**0.96**
6	0.61	1.65	0.26	0.92	6	0.70	1.44	0.19	**0.97**	6	0.74	1.35	**0.15**	**0.97**
HRP1 120 s					HRP1 120 s					HRP1 120 s				
1	0.28	3.53	0.72		few observations					few observations				
2	0.41	2.44	0.48											
3	0.48	2.07	0.38											
4	0.53	1.88	0.33											
5	0.57	1.76	0.29											
6	0.60	1.67	0.27											
HRP1 64 s					HRP1 65 s					HRP1 64 s				
1	0.30	3.38	0.69		few observations					few observations				
2	0.42	2.37	0.46											
3	0.49	2.02	0.37											
4	0.54	1.84	0.32											
5	0.58	1.72	0.28											
6	0.61	1.64	0.26											
HRP2 120s					HRP2 120s					HRP2 120s				
1	0.21	4.66	0.92	0.50	1	0.27	3.76	0.76	0.44	1	0.39	2.59	0.52	0.71
2	0.34	2.97	0.60	0.67	2	0.39	2.55	0.51	0.61	2	0.51	1.96	0.35	**0.83**

3	0.41	2.43	0.48	0.75	3	0.47	2.15	0.41	0.70	3	0.58	1.73	0.29	**0.88**
4	0.46	2.16	0.41	0.80	4	0.52	1.94	0.35	0.76	4	0.62	1.61	0.25	**0.91**
5	0.50	1.99	0.36	0.83	5	0.55	1.81	0.31	**0.80**	5	0.65	1.53	0.22	**0.92**
6	0.53	1.87	0.33	0.86	6	0.58	1.72	0.28	**0.83**	6	0.68	1.48	0.20	**0.94**
HRP2 64 s					HRP2 64 s					HRP2 64 s				
1	0.26	3.89	0.79	0.55	1	0.19	5.22	1.02	0.34	1	0.32	3.09	0.63	0.62
2	0.38	2.61	0.52	0.71	2	0.31	3.22	0.65	0.51	2	0.45	2.22	0.42	0.76
3	0.46	2.19	0.42	0.78	3	0.39	2.60	0.52	0.61	3	0.52	1.92	0.34	**0.83**
4	0.51	1.97	0.36	0.83	4	0.44	2.29	0.44	0.67	4	0.57	1.76	0.29	**0.87**
5	0.54	1.84	0.32	0.86	5	0.48	2.09	0.39	0.72	5	0.60	1.66	0.26	**0.89**
6	0.57	1.74	0.29	0.88	6	0.51	1.96	0.35	0.76	6	0.63	1.58	0.24	**0.91**
