# Supplementary material for: Towards better reliability in fetal heart rate variability using time domain and spectral domain analyses. A new method for assessing fetal neurological state?
Source: PLoS One. 2022 Mar 1;17(3):e0263272. doi: 10.1371/journal.pone.0263272 (PMC8887753; doi:10.1371/journal.pone.0263272)
Supplement: S5 Table — Divided by gestational age. a Gestational age weeks. b 95% Prediction interval within fetus as compared to the true median level as a function of average of n measurements. c Coefficient of variation. d Intraclass correlation coefficient. (PDF) [file pone.0263272.s005.pdf]

GA[a] 20-27					GA[a] 28-34					GA [a]35-41				
n		Within PI[b] (ratio)	Within CV[c]		n		Within PI[b] (ratio)	Within CV[c]		n		Within PI[b] (ratio)	Within CV[c]	ICC[d]
SHRP 120 s					SHRP 120 s					SHRP 120 s				
1	0.39	2.59	0.52	0.76	1	0.50	1.99	0.36	**0.90**	1	0.38	2.60	0.52	**0.83**
2	0.51	1.96	0.35	**0.86**	2	0.62	1.62	0.25	**0.95**	2	0.51	1.97	0.36	**0.91**
3	0.58	1.73	0.29	**0.93**	3	0.67	1.49	0.20	**0.97**	3	0.58	1.74	0.29	**0.95**
4	0.62	1.61	0.25	**0.93**	4	0.71	1.41	0.18	**0.97**	4	0.62	1.61	0.25	**0.95**
5	0.65	1.53	0.22	**0.94**	5	0.74	1.36	0.16	**0.98**	5	0.65	1.53	0.22	**0.96**
6	0.68	1.47	0.20	**0.95**	6	0.76	1.32	**0.14**	**0.98**	6	0.68	1.48	0.20	**0.97**
SHRP64 s					SHRP 64 s					SHRP 64 s				
1	0.30	3.39	0.69	0.60	1	0.41	2.43	0.48	**0.86**	1	0.29	3.49	0.71	0.70
2	0.42	2.37	0.46	0.75	2	0.53	1.87	0.33	**0.92**	2	0.41	2.42	0.48	**0.82**
3	0.49	2.02	0.37	0.82	3	0.60	1.67	0.27	**0.95**	3	0.49	2.06	0.38	**0.87**
4	0.54	1.84	0.32	0.86	4	0.64	1.56	0.23	**0.96**	4	0.54	1.87	0.33	**0.90**
5	0.58	1.73	0.28	0.88	5	0.67	1.49	0.20	**0.97**	5	0.57	1.75	0.29	**0.92**
6	0.61	1.65	0.26	0.90	6	0.70	1.44	0.19	**0.97**	6	0.60	1.67	0.26	**0.93**
HRP1 120 s					HRP1 120 s					HRP1 120 s				
1	0.19	5.19	1.01	0.34										
2	0.31	3.20	0.65	0.51										
3	0.39	2.59	0.51	0.61										
4	0.44	2.28	0.44	0.67										
5	0.48	2.09	0.39	0.72										
6	0.51	1.96	0.35	0.75										
HRP1 64 s					HRP1 64 s					HRP1 64 s				
1	0.15	6.73	1,26	0.10										
2	0.26	3.85	0.78	0.19										
3	0.33	3.01	0.61	0.26										
4	0.39	2.60	0.52	0.31										
5	0.43	2.35	0.46	0.36										
6	0.46	2.18	0.41	0.41										
HRP2 120s					HRP2 120s					HRP2 120s				
1	0.16	6.13	1.16	0.47	1	0.51	1.97	0.36	0,76	1	0.34	2.98	0.60	0.54
2	0.28	3.61	0.73	0.64	2	0.62	1.61	0.25	**0.86**	2	0.46	2.16	0.41	0.70

3	0.35	2.85	0.57	0.73	3	0.68	1.48	0.20	**0.90**	3	0.53	1.88	0.33	0.78
4	0.40	2.48	0.49	0.78	4	0.71	1.40	0.17	**0.93**	4	0.58	1.73	0.28	**0.82**
5	0.44	2.25	0.43	**0.82**	5	0.74	1.35	0.16	**0.94**	5	0.61	1.63	0.25	**0.85**
6	0.48	2.10	0.39	**0.84**	6	0.76	1.32	**0.14**	**0.95**	6	0.64	1.56	0.23	**0.87**
HRP2 64 s					HRP2 64 s					HRP2 64 s				
1	0.06	16.47	2.59	0.14	1	0.26	3.82	0.77	0.49	1	0.26	3.91	0.79	0.40
2	0.14	7.25	1.33	0.25	2	0.39	2.58	0.51	0.66	2	0.38	2.62	0.52	0.57
3	0.20	5.04	0.99	0.33	3	0.46	2.17	0.41	0.75	3	0.46	2.20	0.42	0.67
4	0.25	4.06	0.82	0.40	4	0.51	1.95	0.35	**0.80**	4	0.51	1.98	0.36	0.73
5	0.29	3.50	0.71	0.45	5	0.55	1.82	0.31	**0.83**	5	0.54	1.84	0.32	0.77
6	0.32	3.14	0.64	0.50	6	0.58	1.73	0.28	**0.85**	6	0.57	1.74	0.29	**0.80**
